# Effects of Light and Covering Behavior on *PAX6* Expression in the Sea Urchin *Strongylocentrotus intermedius*

**DOI:** 10.1371/journal.pone.0110895

**Published:** 2014-10-21

**Authors:** Chong Zhao, Nanjing Ji, Ping Sun, Wenping Feng, Jing Wei, Yaqing Chang

**Affiliations:** 1 Key Laboratory of Mariculture & Stock Enhancement in North China's Sea, Ministry of Agriculture, Dalian Ocean University, Dalian, China; 2 College of Marine Life Sciences, Ocean University of China, Qingdao, China; Laboratoire Arago, France

## Abstract

We studied the diel expression pattern of *PAX6* (a structural gene that is commonly involved in the eye development and photoreception of eye forming animals) and the effects of light and covering behavior on *PAX6* expression in the sea urchin *Strongylocentrotus intermedius*. We confirmed that aphotic condition significantly reduced covering behavior in *S. intermedius*. The diel expression pattern of *PAX6* was significantly different in *S. intermedius* under photic and aphotic conditions. The gene expression of *PAX6* significantly deceased in covered *S. intermedius* both under natural light and in darkness. The present finding provides valuable insight into the probable link between covering and *PAX6* expression of sea urchins. Further studies are required to investigate the detailed expression network of light detection involved genes in order to fully reveal the molecular mechanism of the light-induced covering behavior of sea urchins.

## Introduction

The sea urchin is good model for studying the mechanisms of light detection and consequently light-induced behavioral and physiological responses of animals that lack specialized eyes [1]. However, it had remained poorly understood until the genome data of the sea urchin *Strongylocentrotus purpuratus* surprisingly revealed more *opsin* genes and typical “eye” genes (for example, *PAX6*) than previously thought [2]. Recently, it has been convincingly shown that the tube feet are photosensory organs, in which the strong expression of rhabdomeric opsin genes (for example, *Opsin4*) and *PAX6*, highlighted the photoreceptor function and development in the sea urchins *S. purpuratus* [3] and *S. droebachiensis* [4], respectively. *PAX6* is a transcription factor gene commonly involved in eye development and photoreception of eye forming animals. *PAX6* expression indicates conservation of the specification process of the photoreceptor cells (PRCs) consequently deploying as canonical eye genes in echinoids [3]. In addition, PAX6, as a developmental regulator of cell differentiation, directly regulates the expression of opsin genes. However, the responses of *PAX6* expression to environmental and behavioral factors remain largely unknown in sea urchins. Lesser et al. (2011) [4] reported that gene expression of the rhabdomeric-like opsin in the tube feet was significantly different from different areas of the test of *S. droebachiensis*. This suggests that opsin gene expression is related with the amount of exposure of tube feet to light. To our knowledge, however, it is totally unknown whether *PAX6* is expressed in sea urchins in aphotic condition. This greatly hampers our understanding of the complex biological functions of relevant genes in PRCs.

Moreover, we know of no report on the relationship between the relevant expression of PRCs involved genes and the consequent light-induced behavioral response in sea urchins. Covering behavior refers to sea urchins using their tube feet and spines to move objects, such as shells, stones and algal fragments onto their dorsal surface in both shallow water [5] and the deep sea [6]. It has been well documented that covering behavior has evolved as a defensive adaptation against both UV radiation [5], [7], [8] and sunlight intensity [9]. In addition to light responsive functions, opsin genes have recently been shown to have a number of novel functions in *Drosophila*, such as temperature sensation [10] and mechanosensation [11]. It should be noted that covering behavior of sea urchins has been observed in the aphotic deep-sea [6], clearly indicating that light is not the only factor determining covering behavior in sea urchins. This raises an interesting question of whether covering behavior affects expression of PRCs involved genes in sea urchins under aphotic conditions. We hypothesized that covering behavior probably affects the the expression of *PAX6* in sea urchins in both photic and aphotic conditions.

The sea urchin *Strongylocentrotus intermedius* is distributed on intertidal and subtidal rocky bottoms in northern Pacific coastal waters of Hokkaido of Japan, Korea, Far East Russia and China [12]. We were motivated to investigate how light and covering behavior affect the expression of *PAX6* in the sea urchin *Strongylocentrotus intermedius*. The main purposes of the present study are to investigate 1) whether *PAX6* of *S. intermedius* is expressed under short-term aphotic condition; 2) the comparative diel expression patterns of *PAX6* under short-term photic and aphotic conditions; 3) whether aphotic conditions significantly affect covering behavior; 4) whether covering behavior affects *PAX6* expression under both short-term photic and aphotic conditions.

## Results

### *PAX6* expression with exposure to natural sunlight and in darkness

**Figure 1:**
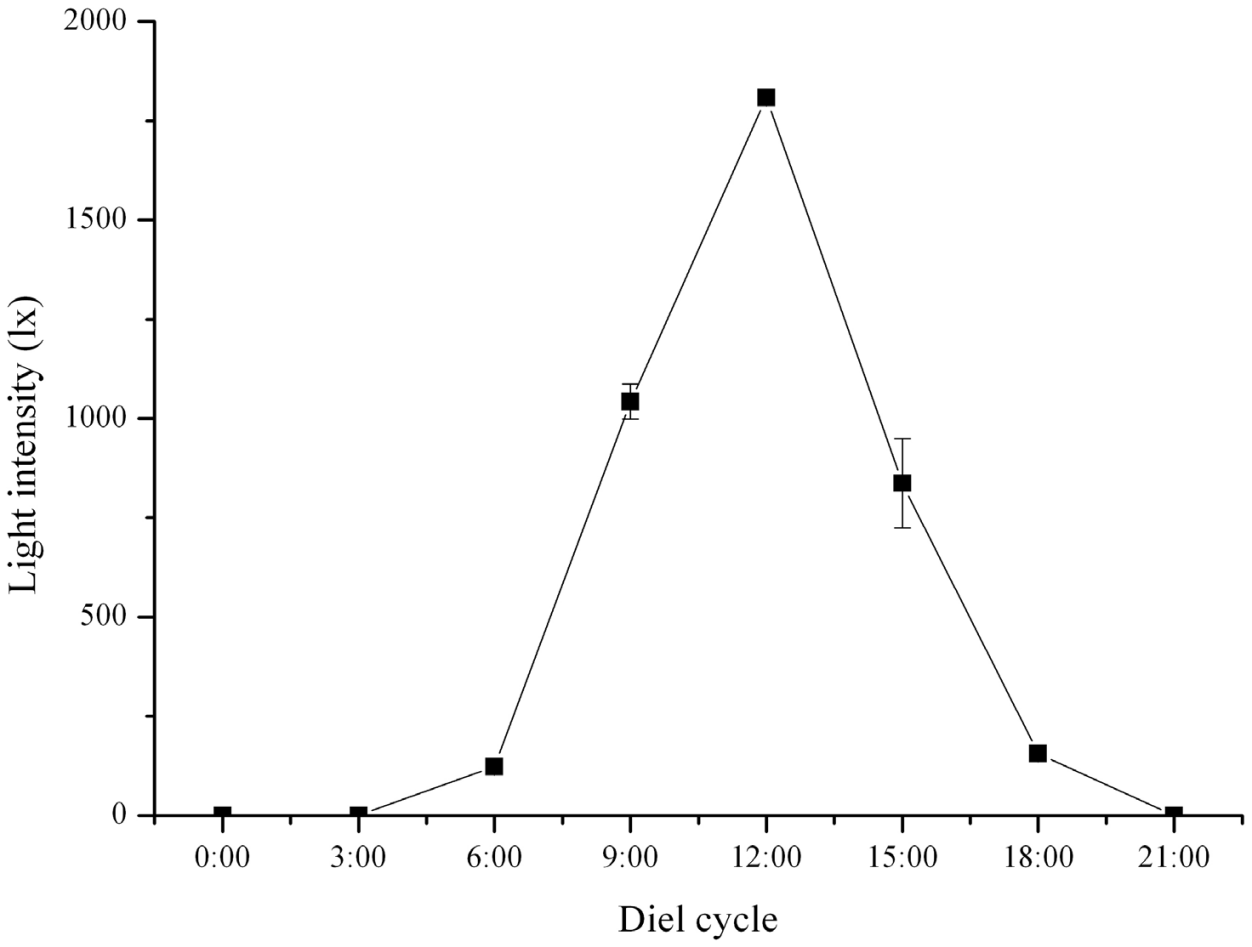
Natural light intensity at the bottom of the tanks during the diel cycle (N = 3, mean ± SE).

Light intensity at the bottom of the tanks was highest at 12:00 and lowest from 21:00 to 3:00 (Fig. 1). The test diameter of all sea urchins used was 35.01±3.09 mm and did not differ significant among light conditions.

**Figure 2:**
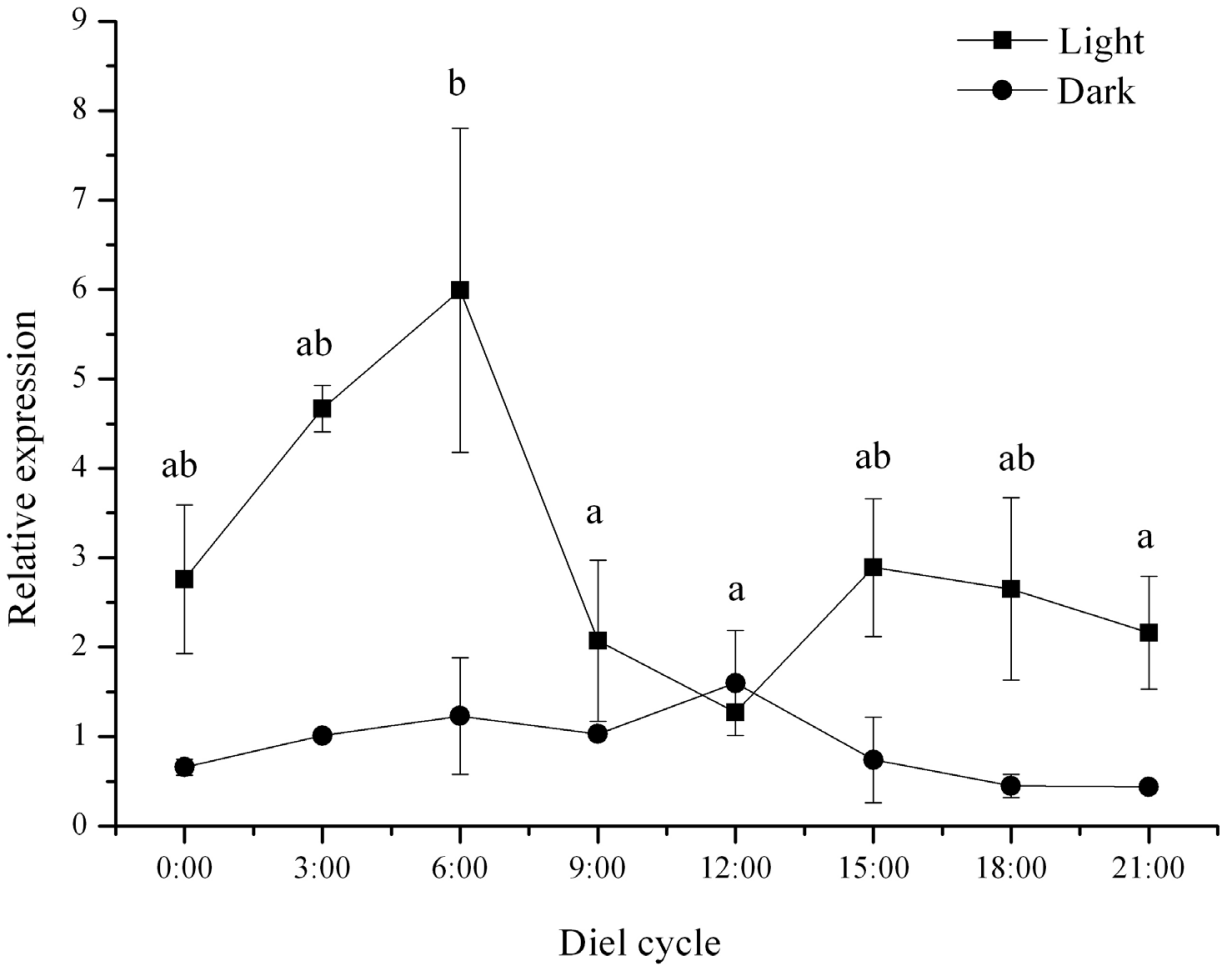
Relative expression of *PAX 6* in tube feet of *Strongylocentrotus intermedius* under different light conditions during a diel cycle (N = 3, mean ± SE). Different letters above the line refer to significant difference in the group of light.

Diel patterns of gene expression of *PAX6* were significantly different between the *S. intermedius* under natural light and dark conditions (*P*<0.001, Fig. 2). *PAX6* of *S. intermedius* in darkness showed a stable relative expression of about 1 during the diel cycle. Relative gene expression of *PAX6* of *S. intermedius* showed a generally inverse relationship to intensity of natural light in the diel cycle, although the *p* was not significant (*P* = 0.071). In this pattern, *PAX6* reached lowest level of relative gene expression at 12:00, when the light intensity was highest in the diel cycle (1808.67±20.70 lx), and significantly increased to the highest level of about 6 at 6:00, when the light intensity was very low (123.73±5.02 lx).

### *PAX6* expression and covering behavior

The mean test diameter of the experimental sea urchins was 40.08±2.38 mm and did not differ significantly among experimental groups (*P*>0.05).

**Figure 3:**
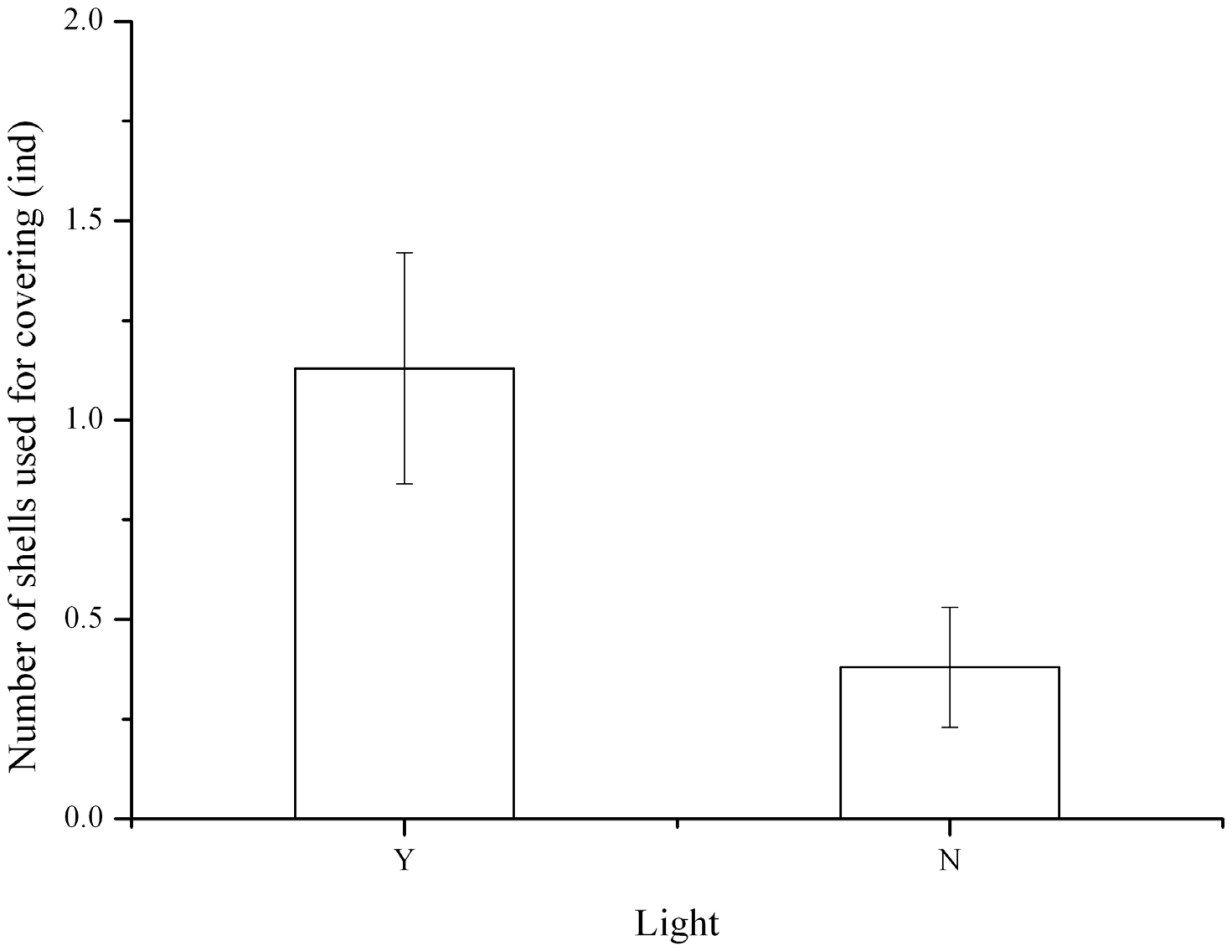
Number of shells used for covering by *Strongylocentrotus intermedius* under photic (Y) or aphotic (N) conditions (N = 16, mean ± SE).

The number of shells used for covering was significantly higher in *S. intermedius* under natural light than in darkness (*P* = 0.029, Fig. 3).

**Figure 4:**
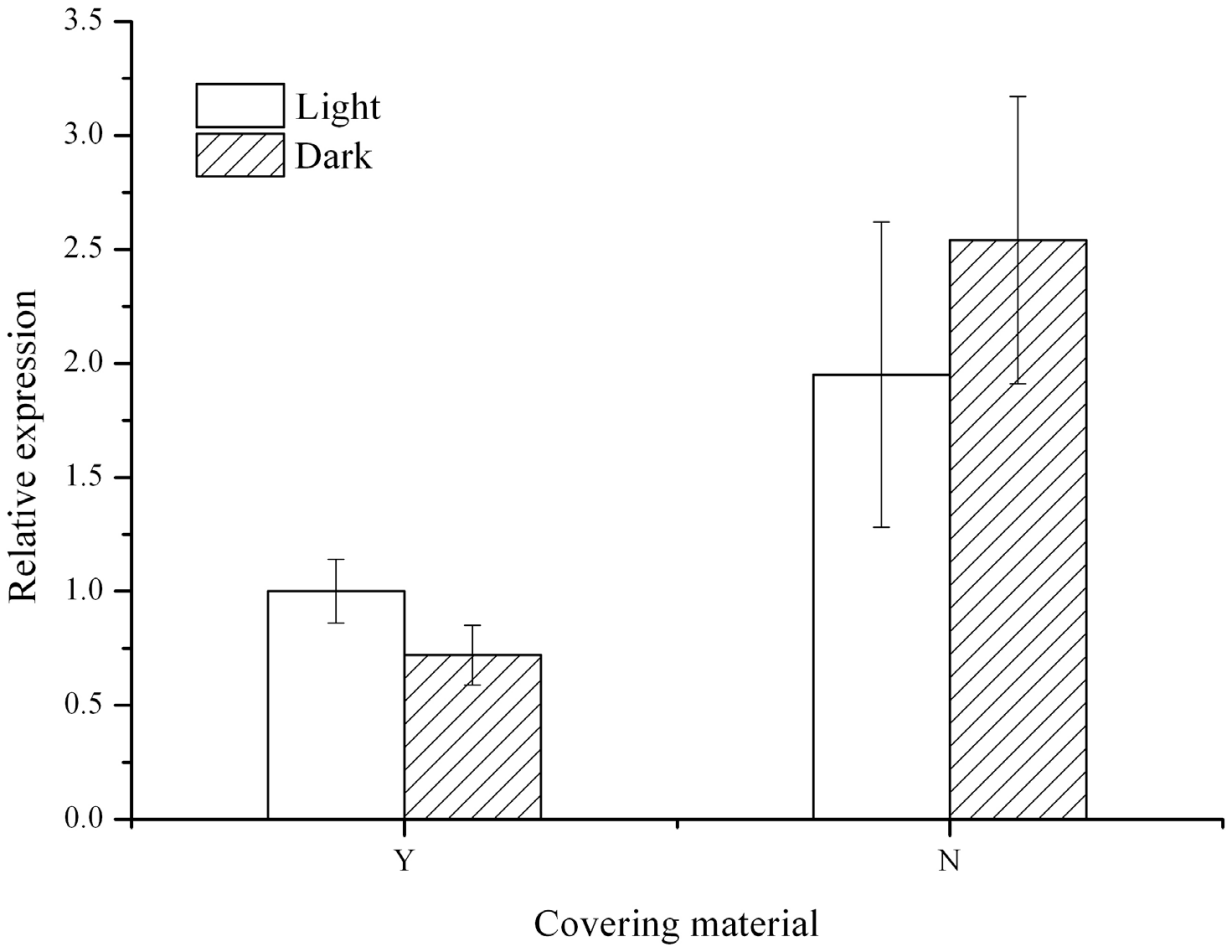
Effect of covering behavior on relative expression of *PAX 6* in *Strongylocentrotus intermedius* under photic and aphotic conditions. Y: covering material present. N: covering material not present. (N = 4, mean ± SE).

Relative expression of *PAX6* significantly decreased in covered *S. intermedius* both under natural light and in darkness (*P* = 0.012, Fig. 4). However, light did not significantly affect the gene expression of *PAX6* (*P* = 0.745).

## Discussion

Light detection by the tube feet of sea urchins involves the arrangement of the ossicles, which function as a light collector [4]. These ossicles are perforated and lined with pigment cells that express PAX6 proteins that are universally involved in the development of eyes in the organisms with eye structures [4]. Understanding the biological functions of *PAX6* in sea urchins remains basically unknown although its pattern of expression has been clearly described [3], [4]. While there is no direct evidence that *PAX6* expression is involved in the light detection of sea urchins, *PAX6* was clearly demonstrated to regulate structural genes involved photoreception in the fruitfly *Drosophila melanogaster* [13]. Thus, it is important to investigate the potential relationship between light condition and *PAX6* gene expression in sea urchins. To our knowledge, the present study is the first investigation on the diel expression pattern of genes involved in light detection in sea urchins. The pattern of expression of *PAX6* in sea urchins under natural light showed a general inverse relationship with the natural light intensity in the diel cycle although the *p* value was not significant. As PAX6 is a direct regulator of the expression of opsin genes in many species [14], a reasonable explanation for the diel pattern of *PAX6* expression is that it translates into the regulation of opsin genes, although we did not measure the expressions of opsin genes in the present study. This opinion is consistent with the previous study by Lesser et al. (2011) [4] of a significant inverse relationship between gene expression of rhabdomeric-like opsin and tube feet position of the test, which involves a difference in exposure to natural light. However, when we put sea urchins in constant darkness, the *PAX6* expression showed a significantly different diel pattern. These results convincingly indicate *PAX6* expression in tube feet of sea urchins is light dependent, although the biological functions remain largely unknown. In the sea urchin *Hemicentrotus pulcherrimus*, Ooka et al. (2010) [15] reported the expression of encephalopsin in the tube feet significantly decreased under dark condition, which is consistent with our result. Thus, it would be very interesting to investigate the expression patterns of *opsin*s and *PAX6* in the sea urchins living in the deep sea [6].

Light has been well documented to be an important factor affecting the covering behavior of sea urchins [7], [8], [9], [16]. In the present study, we found the number of shells used for covering was significantly higher in *S. intermedius* under natural light than in darkness. This result agrees with the finding of Dumont et al. (2007) [7] that sea urchins covered themselves significantly more with exposure to natural light than in darkness. This confirms that light can significantly induce covering behavior in a number of sea urchin species, although covering behavior also exists under aphotic conditions [6]. A generally accepted explanation of this phenomenon is that covering behavior is a protective process against light radiation [9]. However, the molecular neural process of the light-induced covering behavior in sea urchins remains totally unknown. Lesser et al. (2011) [4] hypothesized the light sensitive genes could function in behavioral responses (for example, covering behavior) to light exposure. This raises an interesting question of whether covering behavior significantly affects *PAX6* expression of sea urchins in photic and aphotic conditions. In the present study, we found that covering behavior significantly reduced gene expression of *PAX6* in *S. intermedius* under natural light. As covering behavior, as generally accepted, has a protective function against light radiation, covered sea urchins should detect less light than uncovered sea urchins. However, the diel pattern result of the *PAX6* expression showed that expression of *PAX6* significantly increased in sea urchins when light intensity decreased. It is unreasonable to suppose that covering behavior does not protect sea urchins against light radiation because covered sea urchins have their test covered and tube feet retracted. This is supported by our further finding that the *PAX6* expression of *S. intermedius* also significantly decreased in covered sea urchins in darkness. These results clearly indicate that the significant effect of covering behavior on *PAX6* expression is not light dependent under the experimental conditions. It also highlights that *PAX6* of sea urchins probably plays complex and novel biological roles, which have been well documented in vertebrates. For example, Kim et al (2014) [17] found that *PAX6* expression played an important role in glutamatergic neuronal differentiation, which effectively regulated the autism-like behavior of rats, whose parents were prenatally exposed to valproic acid. These results suggest that there is probably a novel link between *PAX6* expression and covering in sea urchins, especially in the species existing in aphotic deep seas [6]. Further studies are required to investigate the detailed expression network of relevant genes involved in light detection, in order to completely reveal to the molecular mechanism of the light-enhanced covering behavior of sea urchins.

In conclusion, we confirmed that aphotic condition significantly reduced covering behavior in *S. intermedius*. The diel expression pattern of *PAX6* was significantly different in *S. intermedius* under short-term photic and aphotic conditions. The gene expression of *PAX6* significantly deceased in covered *S. intermedius* both under short-term natural light and in darkness. The present study enriches our understanding about the responses of *PAX6* expression to light and covering behavior in sea urchins. To be noted, however, the present study did not measure the protein level of PAX6. This should be investigated in the future.

## Materials and Methods

### Sea urchins

A batch of *S. intermedius* was transported from Dalian Haibao Fisheries Company to Key Laboratory of Mariculture & Stock Enhancement in North China's Sea, Ministry of Agriculture, Dalian Ocean University on July 15, 2013. They were maintained in tanks and fed kelp ad libitum for 10 days to acclimate the natural cycle until used in the experiment.

### *PAX6* expression with exposure to natural sunlight and in darkness

Natural sunlight and darkness were set up as the two light conditions. The intensity of natural sunlight was measured at the bottom of the tank during the diel cycle with an underwater irradiance meter. The tanks of the darkness group were covered with black plastic cloth to exclude light. Nine sea urchins were haphazardly collected from both light conditions at 0:00, 3:00, 6:00, 9:00, 12:00, 15:00, 18:00 and 21:00 hours on July 25 and 26, 2013. They were placed into containers with seawater to relax and extend their tube feet. There was no exposure to light except for quick samplings of urchin in darkness under very dim artificial light. Tube feet from the aboral portion of the test were quickly collected in from each individual. Tube feet from three individuals were combined and immediately frozen in liquid nitrogen and stored at −80°C until analysis.

### *PAX6* expression and covering behavior

The same conditions were set up as in the above section. Twenty shells of the small bivalve *Patinopecten yessoensis* (shell length: 21.38±1.28 mm, shell height: 19.88±1.60 mm, shell weight: 0.29±0.07 g) were used as potential covering material and distributed evenly on the bottoms of the eight experiments tanks (55 cm×44 cm×37 cm). Eight similar tanks without covering material were set up as controls. The experiment began at 9:00, and ended at 12:00 on September 18, 2013. The number of shells used for covering was recorded 3 hours after the beginning of the experiment. The tube feet were collected as in the above section.

### Total RNA extraction and cDNA synthesis

Total RNA was extracted from the tube feet of *S. intermedius* with the RNAsimple total RNA kit (TIANGEN, China) following the manufacturer instructions. The concentration and quality were determined using the NanoPhotometer (Implen GmbH, Germany) and agarose gels electrophoresis. First stand cDNA was synthesized using the RT reagent Kit (TaKaRa, Japan) according to the manufacturer instructions.

### *PAX6* expression measurement by quantitative Real-time PCR

The expression of *PAX6* mRNA in *S. intermedius* was studied by quantitative Real-time PCR (qRT-PCR). The gene-specific primers were designed based on the cloned cDNA fragment of *S. intermedius PAX6* (KF733999) using Primer Premier 5 software (Table 1). 18S ribosomal RNA was used as the reference gene [18]. The qRT-PCR was carried out in a 20 µL volume including 10 µL of 2×SYBR Green Master mix (TaKaRa, Japan), 0.4 µL ROX Reference Dye II, 0.4 µM of each primer, 1 µL of 1∶3 diluted cDNA and 7 µL ddH_2_O using the Applied Biosystem 7500 Real-time system (Applied Biosystem, USA). The qRT-PCR profile was as follows: 95°C for 30 s, followed by 40 cycles of 95°C for 5 s and 60°C for 32 s. At the end of each PCR reaction melting curve analysis of amplication products was preformed to confirm amplification specificity. The relative mRNA levels of the target genes were calculated as 2^−ΔΔCT^ method [19].

**Table 1:** PCR primers used in the study.

Primer names	Sequence (5′→3′)	Purpose
*PAX6*-F	CTACGAGACGGGGAGCATA	For *PAX6* qRT-PCR
*PAX6*-R	CCCTCTTGTAGTGGGCAAT	
18S-F	GTTCGAAGGCGATCAGATAC	For qRT-PCR
18S-R	CTGTCAATCCTCACTGTGTC	

### Statistical analysis

Diel patterns of relative expression of *PAX6* and effect of covering behavior were analyzed by two-way ANOVA. The number of shells used for covering in the two treatments was tested by one-way ANOVA. Correlations between intensity of light and *PAX6* expression were analyzed in both males and females using Pearson correlation analysis. All analyses were performed with SPSS 13.0 statistical software. A probability level of *P*<0.05 was considered statistically significant.
